# Tubercular Adenitis—Treatment by X-Rays

**Published:** 1907-04

**Authors:** 


### SELECTIONS AND ABSTRACTS.

### TUBERCULAR ADENITIS—TREATMENT BY X-RAYS.

The results of twelve cases are reported by Barjon (Lyon Medicale, No. 41, 1906), who found the principal effect on the general infiltration which so often accompanies scrofula, uniting the lymph glands in a solid mass. The glands become separated soon after beginning the applications, and later disappear. If there is any tendency to softening the rays hasten this, and the abscess soon requires to be opened. The rays continue to have a good effect on the suppurating tissues. No bad effects or tendency to cause metastasis were noted.

